# Polyserositis as a primary clinical manifestation of CD7+ acute myelogenous leukemia with myeloid sarcoma: A case report

**DOI:** 10.1097/MD.0000000000023615

**Published:** 2020-12-11

**Authors:** Yubo Pi, Beining Wang, Lihong Wang, Hanyun Ren

**Affiliations:** Department of Hematology, Peking University First Hospital, Peking University, Beijing, China.

**Keywords:** acute myeloid leukemia, case report, myeloid sarcoma, polyserositis

## Abstract

**Rationale:**

Myeloid sarcomas (MS) are defined as rare extramedullary masses composed of immature myeloid cells. MS mostly develops in patients with acute myeloid leukemia (AML), and involves primarily the skin, soft tissues, bones, and lymph nodes. Pleura and pericardium involvement of MS are extremely uncommon. Polyserositis is also a very rare extramedullary presentation of acute myeloid leukemia (AML).

**Patient concerns:**

A 30-year-old woman with a complaint of right neck mass combined with coughing for 2 months as well as fever and systemic edema for the last 10 days, was admitted to our center on July 11, 2019. Initial positron emission tomography (PET) scan indicated systemic lymphadenopathy, bilateral pleural effusion, and pericardial effusion.

**Diagnosis:**

The initial pathological diagnosis of lymph nodes was MS. Subsequent bone marrow analysis confirmed AML.

**Interventions:**

Conventional IA induction regimen followed by high-dose cytarabine (HiDAC) regimen.

**Outcomes:**

Complete absorption of pericardial and pleural effusion after the first cycle of IA induction chemotherapy.

**Lessons:**

Polyserositis can be an extramedullary presentation of AML. Patients with polyserositis should undergo routine flow cytometric analysis. For AML with extamedullary infiltration, systemic chemotherapy should be administered in all confirmed cases.

## 1 Introduction

Myeloid sarcoma (MS) is a rare form of localized malignancy that is caused by extramedullary proliferation and infiltration of myeloid primitive cells and immature myeloid cells.^[1]^ This type of sarcoma is most frequently associated with AML, but can also develop from chronic myeloid leukemia (CML) and myeloproliferative neoplasm (MPN).^[2,3]^ Regarding its association with AML, MS can occur prior to or concurrently with AML, or as a form of extramedullary relapse. Most commonly, MS involves the skin, soft tissues, bones, and lymph nodes. Polyserositis as a result of leukemic infiltration, on the other hand, is an extremely rare extramedullary presentation of AML with only one case documented to date.^[4]^ In this report, we present a case of acute myelogenous leukemia with myeloid sarcoma confirmed in the lymph nodes. Notably, AML also manifests as polyserositis.

## 2 Case report

**Figure 1:**
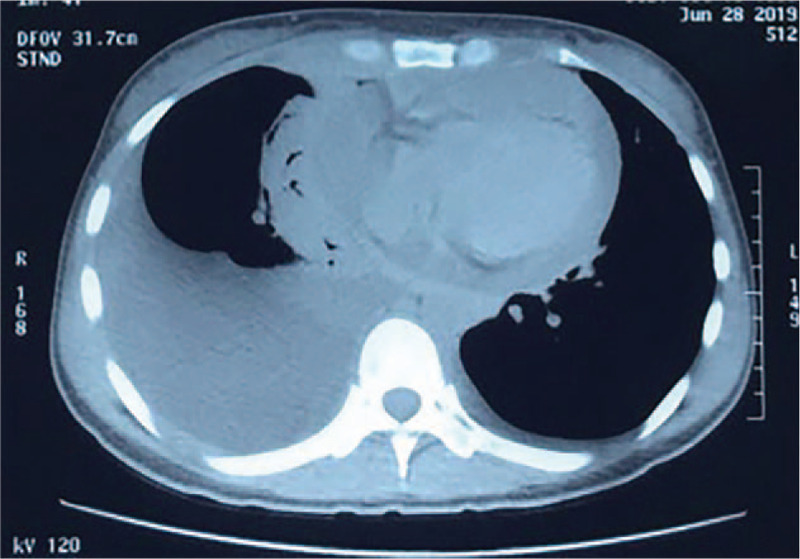
Initial chest CT.

**Figure 2:**
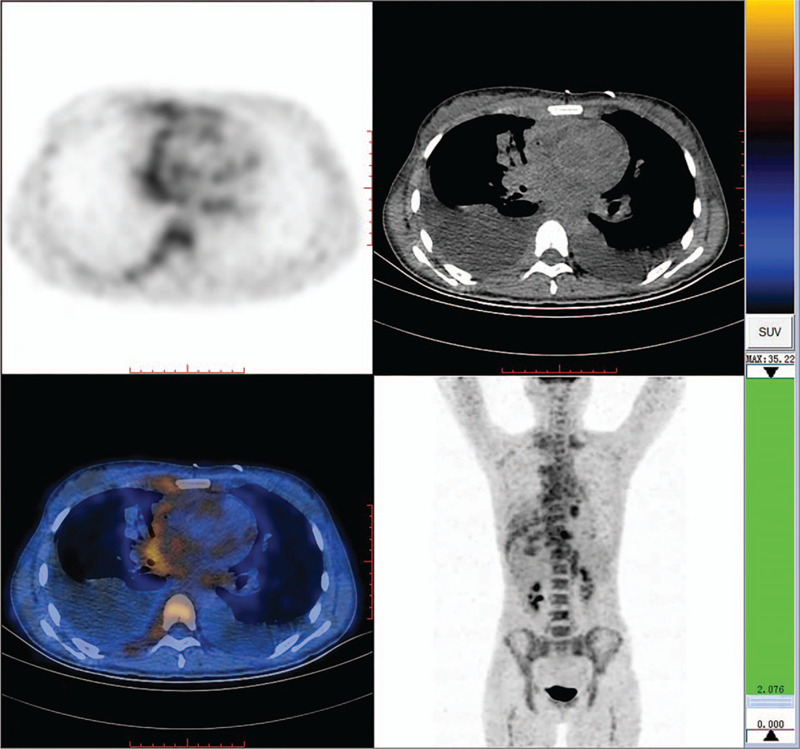
Initial PET scan.

A 30-year-old woman with a complaint of right neck mass combined with coughing for 2 months as well as fever and systemic edema for the last 10 days, was admitted to our center on July 11, 2019. The patient was diagnosed with a systemic infection. Prior to admission, the patient suffered from acute pericardial tamponade that required emergency pericardiocentesis. Otherwise, she was previously healthy. Her initial chest CT is shown below in Figure 1 and the initial positron emission tomography (PET) scan in Figure 2. As suggested by the PET scan, increased diffuse uptake of ^18^F-FDG in the spine, pelvis, and femur was detected. Additionally, systemic lymphadenopathy with a maximum standard uptake value (SUV_max_) of 6.6, thickening region of the right pleura with SUV_max_ of 4.3, and bilateral pleural effusion and pericardial effusion were also evident.

**Figure 3:**
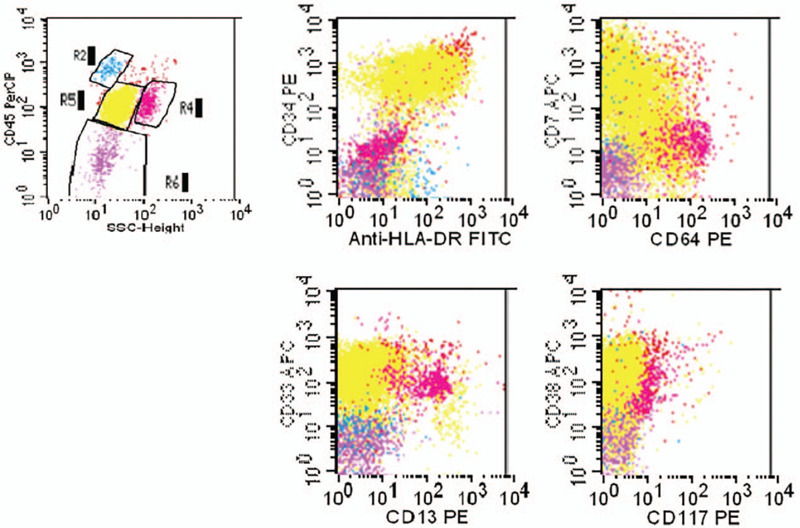
Flow cytometric analysis of bone marrow aspirate. R5 were myleoblasts positive for CD7, CD34, CD33, CD38, HLA-DR.

Laboratory tests indicated a white blood cell count of 16.4 × 10^9^/L, with blasts accounting for 83%. Tests on bone marrow aspirate indicated a myeloblast proportion of 79.5%. Flow cytometric analysis revealed that the bone marrow blasts were positive for CD7, CD34, CD33, CD38, and HLA-DR, and negative for CD117, CD3, CD20, CD10, CD19, CD56, CD14, CD64, CD11b, CD13, GlyA, CD7, CD61, cMPO, cCD3, cCD79a, cCD22, and cTDT, confirming a diagnosis of AML (Fig. 3). Cytogenetics revealed no abnormalities, and genotyping was negative for MLL, EV11, FLT3, NPM1, and C-KIT.

**Figure 4:**
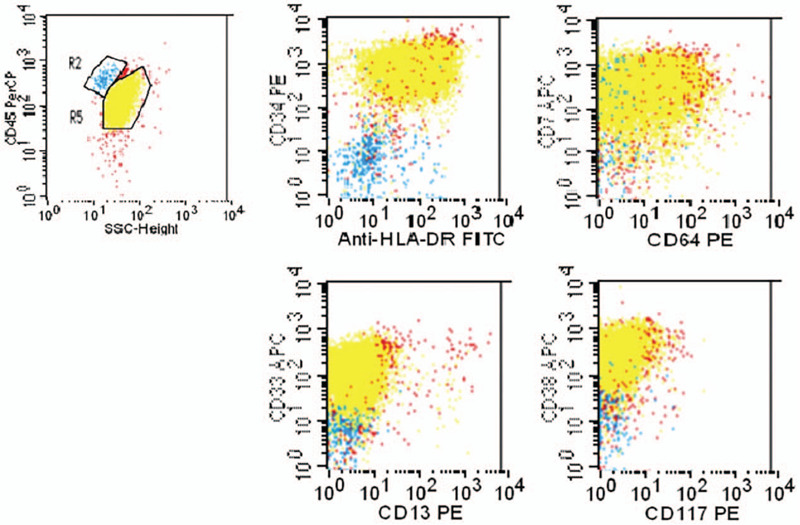
Flow cytometric analysis of pleural effusion.R5 were myleoblasts positive for CD7, CD34, CD33, CD38, HLA-DR.

**Figure 5:**
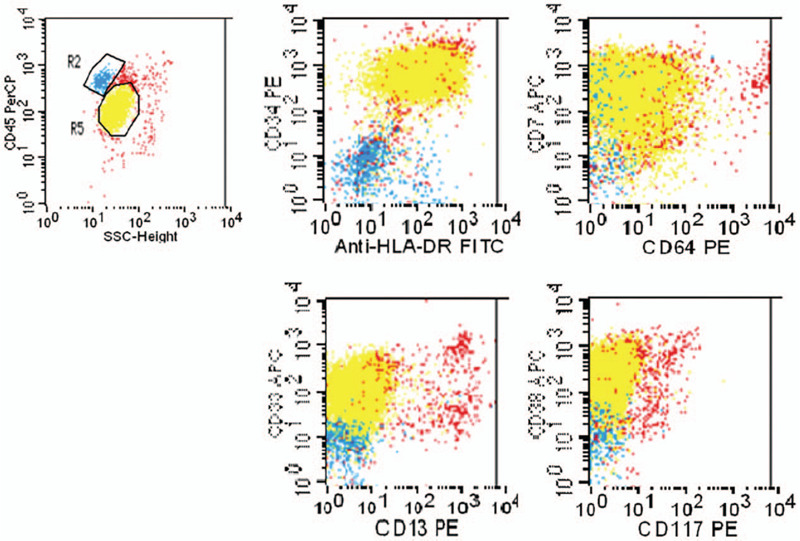
Flow cytometric analysis of pericardial effusion.R5 were myleoblasts positive for CD7, CD34, CD33, CD38, HLA-DR.

Thoracentesis and pericardiocentesis were performed, and both pleural and pericardial fluid turned out exudative according to Light's criteria. Flow cytometric analysis revealed that both effusions were predominantly myeloblasts, with an immunophenotype identical to bone marrow blasts (Figs. 4 and 5). Thus, a diagnosis of AML involving the pleura and pericardium was confirmed. Pathological examination of her axillary lymph node indicated infiltration of primitive cells, of which immunohistochemical staining was positive for MPO, TdT, CD68, CD34, CD99, and lysozyme (sporadic), while Ki-67 was expressed in 80% of the cells. Therefore, the diagnosis was further confirmed as myeloid sarcoma.

**Figure 6:**
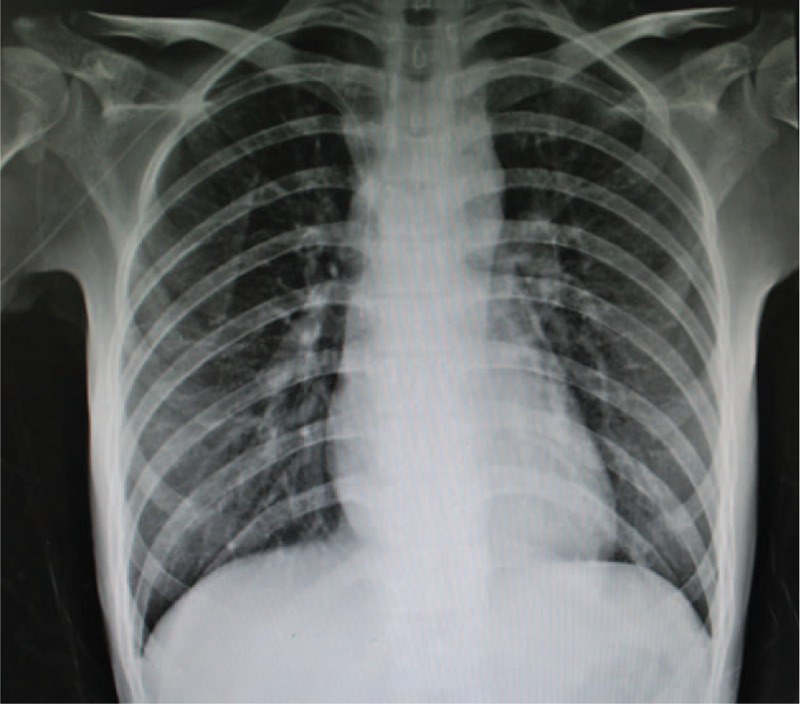
Follow-up x-ray after the first cycle of IA induction chemotherapy.

**Figure 7:**
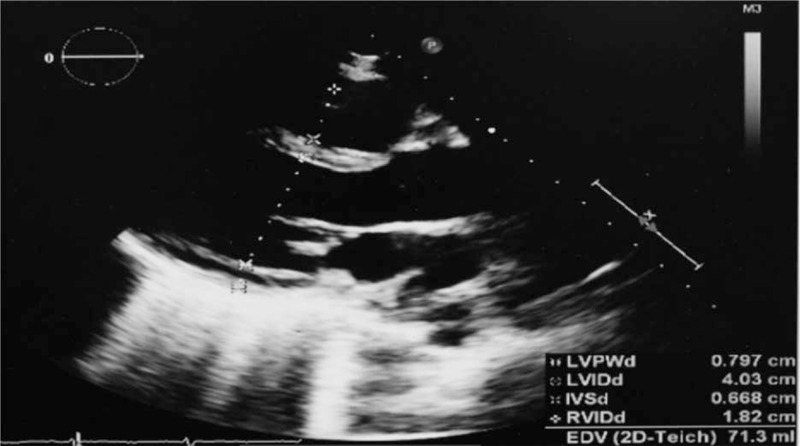
Follow-up echocardiography after the first cycle of IA induction chemotherapy.

According to National Comprehensive Cancer Network guidelines, the patient subsequently received a conventional 3 + 7 induction regimen: 125 mg/m^2^ cytarabine from D1 to D7, and 12 mg/m^2^ of idarubicin from D1 to D3. Follow-up echocardiography and chest X-ray indicated complete absorption of pericardial and pleural effusion (Figs. 6 and 7). Bone marrow aspirate indicated 23.4% myeloblasts. Afterwards, she received high-dose cytarabine (HiDAC) regimen, with 3 g/m^2^ of cytarabine every 12 h from D1 to D6. Following chemotherapy, the patient developed pneumonia and diffuse alveolar hemorrhage. Broad-spectrum antibiotics (piperacillin-tazobactam, meropenem, linezolid, and tigercycline), antifungals (amphotericin B and cancidas), and hemostasis drugs (pituitrin, carbazochrome sodium sulfonate, and hemocoagulase) were administered. Eventually, she refused further treatments and died 69 days after the initiation of induction chemotherapy.

## 3 Discussion

MS as extramedullary infiltration of immature myeloid cells is a very rare condition. It mostly develops in patients with AML. Previous reports have suggested no significant difference between the prognosis of isolated MS and MS with concomitant AML.^[5–7]^ According to a recent study, approximately 55% of MS cases also expressed CD7.^[5]^ CD7-postive AML exhibited high blast cell counts and poor outcomes.^[8–10]^ Additionally, it has been reported that leukemic pleural effusion could also be an initial manifestation of CD7-positive AML.^[11]^

As far as pleural effusion is concerned, it can occasionally occur during the clinical course of any hematological malignancies, especially in Hodgkin and non-Hodgkin lymphomas.^[12]^ However, leukemic pleural effusion is rare, and no more than 20 cases have been reported.^[4,11,13–26]^ The average age of patients reported in the literature was 53 years (range, 22–76 years), of whom 14 were male and 4 were female. One of these cases was CD7 positive AML. However, the exact incidence rate of leukemic pleural effusion is difficult to estimate. Awasthi et al found that 18% of 898 pleural fluid samples were malignant, yet none of them were AML.^[27]^ Cakir et al reviewed 4684 pleural fluid specimens in Turkey and found only one case of AML.^[28]^ Although the prognostic implication of leukemic pleural effusion in AML is undetermined, most cases have a poor prognosis. Nevertheless, both induction chemotherapy and stem cell transplantation were proven efficacious against this particular condition.^[11,17]^

In contrast, pericardial effusion can occur in 21% of patients with AML at initial diagnosis, but only 0.5% manifests as pericardial tamponade,^[29]^ a condition for which only 4 documented cases can be found.^[30–33]^ The average age of these 4 patients was 42 years (range, 28–73 years), of whom 3 were male and 1 was female. 2 of 4 patients died prior to any possible treatment. Therefore, the prognostic value of leukemic pericardial effusion needs further investigation. Nevertheless, early recognition may improve clinical outcomes.

In addition to pleural and pericardial effusion, our case of MS also involved lymph nodes. MS can be the initial manifestation of AML, particularly for M5 (monocytic) and M4 (myelomonocytic) subtypes.^[34,35]^ Treatments for MS include systemic chemotherapy, surgical resection, and local radiotherapy.^[36]^ Anti-AML induction remission therapy is sufficient and effective for MS.^[2,35]^ Our patient received IA and HiDAC induction therapy according to AML protocols, which resulted in complete resolution of pericardial and pleural effusion. However, complete remission (CR) was not achieved in the bone marrow, indicating different biological characteristics from extramedullary infiltration. Since the patient refused further examination, a definite validation regarding the potential difference was no longer possible.

In conclusion, although polyserositis as a primary clinical manifestation of AML is extremely rare, patients with polyserositis should still undergo routine flow cytometric analysis in order to avoid delay in diagnosis. For AML with extramedullary infiltration, systemic chemotherapy should be administered in all confirmed cases.

## Author contributions

**Data curation:** Yubo Pi, Beining Wang.

**Supervision:** Lihong Wang, Hanyun Ren.

**Writing – original draft:** Yubo Pi.

**Writing – review & editing:** Lihong Wang, Hanyun Ren.
